# The Safety and Efficacy of Live Viral Vaccines in Patients With Cartilage-Hair Hypoplasia

**DOI:** 10.3389/fimmu.2020.02020

**Published:** 2020-08-11

**Authors:** Svetlana Vakkilainen, Iivari Kleino, Jarno Honkanen, Harri Salo, Leena Kainulainen, Michaela Gräsbeck, Eliisa Kekäläinen, Outi Mäkitie, Paula Klemetti

**Affiliations:** ^1^Children’s Hospital, Pediatric Research Center, University of Helsinki and Helsinki University Hospital, Helsinki, Finland; ^2^Folkhälsan Research Center, Institute of Genetics, Helsinki, Finland; ^3^Research Program for Clinical and Molecular Metabolism, Faculty of Medicine, University of Helsinki, Helsinki, Finland; ^4^Translational Immunology Research Program, University of Helsinki, Helsinki, Finland; ^5^Children’s Hospital, Clinicum, University of Helsinki, Helsinki, Finland; ^6^Department of Pediatrics and Adolescents, Turku University Hospital, University of Turku, Turku, Finland; ^7^Department of Pediatrics, Kymenlaakso Central Hospital, Kotka, Finland; ^8^Helsinki University Hospital, HUSLAB, Division of Clinical Microbiology, Helsinki, Finland; ^9^Department of Molecular Medicine and Surgery and Center for Molecular Medicine, Karolinska Institutet, Stockholm, Sweden; ^10^Department of Clinical Genetics, Karolinska University Hospital, Stockholm, Sweden

**Keywords:** clinical trial, combined immunodeficiency, immunization, MMR, RMRP, vaccination, varicella zoster virus

## Abstract

**Background:**

Live viral vaccines are generally contraindicated in patients with combined immunodeficiency including cartilage-hair hypoplasia (CHH); however, they may be tolerated in milder syndromes. We evaluated the safety and efficacy of live viral vaccines in patients with CHH.

**Methods:**

We analyzed hospital and immunization records of 104 patients with CHH and measured serum antibodies to measles, mumps, rubella, and varicella zoster virus (VZV) in all patients who agreed to blood sampling (*n* = 50). We conducted a clinical trial (ClinicalTrials.gov identifier: NCT02383797) of live VZV vaccine on five subjects with CHH who lacked varicella history, had no clinical symptoms of immunodeficiency, and were seronegative for VZV; humoral and cellular immunologic responses were assessed post-immunization.

**Results:**

A large proportion of patients have been immunized with live viral vaccines, including measles-mumps-rubella (MMR) (*n* = 40, 38%) and VZV (*n* = 10, 10%) vaccines, with no serious adverse events. Of the 50 patients tested for antibodies, previous immunization has been documented with MMR (*n* = 22), rubella (*n* = 2) and measles (*n* = 1) vaccines. Patients with CHH demonstrated seropositivity rates of 96%/75%/91% to measles, mumps and rubella, respectively, measured at a medium of 24 years post-immunization. Clinical trial participants developed humoral and cellular responses to VZV vaccine. One trial participant developed post-immunization rash and knee swelling, both resolved without treatment.

**Conclusion:**

No serious adverse events have been recorded after immunization with live viral vaccines in Finnish patients with CHH. Patients generate humoral and cellular immune response to live viral vaccines. Immunization with live vaccines may be considered in selected CHH patients with no or clinically mild immunodeficiency.

## Introduction

Cartilage-hair hypoplasia (CHH, MIM #250250) is a rare skeletal dysplasia with combined immunodeficiency. CHH is caused by mutations in the *RMRP* gene, encoding the RNA component of the mitochondrial RNA-processing endoribonuclease RNase MPR (1). Impaired *RMRP* functioning results in cell cycle disturbances, altered telomere biology and changes in gene regulation (2–4). Patients with CHH demonstrate highly variable degree of immune defect, ranging from asymptomatic lymphopenia to severe combined immunodeficiency necessitating hematopoietic stem cell transplantation (5, 6).

The first report on CHH among the Amish described fatal outcomes after varicella zoster virus (VZV) primary infection (7). Since then, several CHH patients with severe varicella, but not fatalities, have been reported (8–12). In the large Finnish case series published in 1990s, only two out of 56 patients with a history of varicella had required hospitalization (13). In a more recent Amish series of 25 patients, neither antiviral medications nor hospitalizations were needed for varicella (14).

Patients with CHH can develop fatal complications after administration of live viral vaccines against smallpox or polio (15, 16). The development of humoral or cellular response after live vaccine administration has not been previously assessed in CHH. CD4+ cells producing interferon gamma (IFN-γ) contribute significantly to the immunity from VZV, and measurement of IFN-γ response represents a simple method for evaluation of VZV-specific cellular immunity (17, 18).

Live vaccines are generally contraindicated in patients with combined immunodeficiency, although they can be tolerated in milder syndromes (19). Measles-mumps-rubella (MMR) and VZV vaccines are safe in children with partial DiGeorge syndrome who have CD4 + T cell counts of ≥500 cells/mm^3^ (19). In children infected with human immunodeficiency virus, live vaccines are considered safe if CD4 + T cell count is >200 cells/mm^3^ or >15% (19).

In order to enable evidence-based decisions on the immunization of patients with CHH, we collected clinical data on the course of live-vaccine preventable diseases and analyzed vaccination and serologic data for the administered live viral vaccines in a large cohort of Finnish CHH patients, and then conducted a clinical trial with live VZV vaccine in this patient population.

## Materials and Methods

The study protocol and the clinical trial (ClinicalTrials.gov identifier: NCT02383797, registered on March 9, 2015) were approved by the Institutional Ethics Committee at Helsinki University Hospital and University of Helsinki, Finland. Additional approval for the clinical trial was obtained from the Finnish Medicines Agency (FIMEA). The study was conducted in accordance with the Declaration of Helsinki and national and institutional standards. All participants and/or caregivers signed informed consents.

### Clinical and Laboratory Data

Clinical data, including data on live-vaccine preventable diseases, were obtained from the patients directly, as well as retrospectively from health records, as part of our previous studies exploring characteristics and natural course of CHH in the Finnish cohort (5, 13, 20, 21). Only provider-recorded data in the patients’ immunization certificates were considered reliable and were included in the analysis of immunizations. The researchers had access to data on all hospitalizations of the study patients, beginning from 1969, via the Health Registers described in detail previously (21).

Serum samples were obtained from patients during clinical visits as part of our previous study (5). All patients who agreed to blood sampling were included, except those with ongoing immunoglobulin replacement therapy. The patients were not selected on the basis of the history of live-vaccine preventable diseases or immunization with live vaccines. Samples were analyzed by enzyme immunoassays for the presence of antibodies to measles, mumps, rubella, and VZVs. Reference values of the local laboratory (HUSLAB) were applied, and the titers were considered protective if over 15 EIU for rubella and VZVs, while for antibodies against measles and mumps viruses, qualitative assays were used.

### Clinical Trial of Live Varicella Vaccine

**FIGURE 1:**
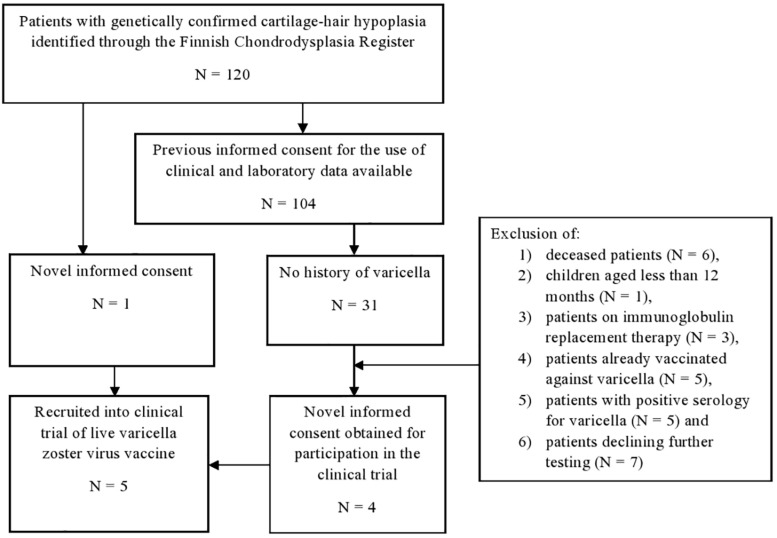
Recruitment to the clinical trial of safety and efficacy of live varicella zoster virus vaccine in patients with cartilage-hair hypoplasia.

We conducted an open-label clinical trial on live VZV vaccine (Figure 1). Of the 120 patients with CHH in the Finnish Skeletal Dysplasia Register, we recruited five patients. Inclusion criteria were: (1) genetically confirmed CHH, (2) age >12 months, and (3) no history of varicella. Exclusion criteria were (1) history of immunization against VZV, (2) positive serum antibodies for VZV, (3) low CD4 + T cell counts (<15% or <200 cells/mm^3^), (4) clinical signs of severe immunodeficiency, or (5) ongoing immunoglobulin replacement therapy.

In 2015–2017, all recruited patients were given one or two doses of Varilrix^®^, a live-attenuated VZV Oka strain vaccine, subcutaneously, by the nurses in the health care facilities. One dose contained at least 10^3^.^3^ plaque forming units of VZV. A second dose was administered if the serologic response after primary immunization was negative.

Blood samples were drawn pre-immunization and 4-6 weeks post-immunization for evaluation of humoral and cellular responses. Humoral responses were assessed by measurement of serum VZV-specific antibodies by enzyme immunoassay in HUSLAB. Cellular responses were assessed by elispot using cryopreserved peripheral blood mononuclear cells (PBMC). Control PBMC samples were obtained from (1) healthy adult volunteers with a history of varicella disease, (2) CHH families, healthy siblings who had been immunized against varicella, and 3) one patient with CHH and a history of varicella.

Peripheral blood mononuclear cells were isolated from heparinized blood samples by Ficoll centrifugation. The isolated cells were counted and frozen in RPMI1640 cell culture medium containing 10% heat-inactivated human male serum (Innovative Research, Peary Court Novi, MI, United States) and 10% Dimethyl sulfoxide. Freezing of the isolated PBMCs to −80°C was done gradually in a temperature-controlled way in 1.8 ml cryotubes using isopropanol-filled cryofreezing containers. The next day the frozen cells were transferred to automated liquid nitrogen freezer (AGA, Finland) for long term storage at −180°C until use.

Peripheral blood mononuclear cells from patients with CHH and controls were subjected to IFN-γ elispot antigen response analysis (3420-4HST-2, MabTech, Nacka Strand, Sweden). The elispot was performed according to manufacturer’s protocol with 24 h stimulation with VZV antigen preparation (PR-BA104, Jena Bioscience, Löbstedter, Germany). Shortly, thawed PBMC were cultured on IFN-γ elispot strips in CTL-test medium (CTLT-010, Cellular Technology Limited, OH, United States) in 5% CO_2_ humidified incubator at 37°C. For antigen response, cells in triplicate wells were stimulated with 2 ug of VZV antigen and compared to CTL-test only negative controls. MabTech anti-CD3 (1:1000) was used as positive control to TCR-stimulant for T cells. The IFN-γ spots were made visible with streptavidin-HRP enzymatic assay (3420-4HST-2, MabTech) and the number of IFN-γ spots were counted with elispot reader system (Elispot Reader System ELRIFL04, AID, Strassberg, Germany) with cutoff settings for a size and staining intensity of the positive spots deriving from the corresponding positive and negative control spots. The identity of patient and control samples was blinded during the elispot analysis. The statistical analysis of the elispot data (Paired-Wilcoxon) was done in RStudio (1.25) running R (3.6.2) with packages (rstatix, ggpubr, and tidyverse) available in r-project (cran.r-project.org). If patient had received two doses of vaccine, the data from the sample collected after the second dose was used in the analysis. One sample (vaccinated healthy control) was dropped from the data due to very low level of reactive T cells in anti-CD3 positive control.

All patients/caregivers were instructed to report any adverse reactions immediately to the researchers and were additionally contacted by phone 4–6 weeks post-immunization to record potential adverse events. In addition, patient records of all clinical trial participants were assessed at the time of writing (3–5 years post-immunization) to check for possible long-term adverse events or breakthrough VZV infection.

## Results

### Cohort Description

Of the identified 120 patients with CHH in Finland, informed consent for the use of clinical and laboratory data was obtained from 104 patients during our previous studies (Figure 1). All patients were either homozygous or compound heterozygous for the g.71A > G *RMRP* mutation.

The clinical characteristics of this cohort have been reported in our recent publication (20), According to our clinical immunodeficiency categorization (21) and based on data on childhood symptoms available in 103/104 patients, 62 patients were classified as clinically asymptomatic, another 28 individuals demonstrated symptoms of humoral immunodeficiency with recurrent respiratory tract infections and/or sepsis, and the remaining 13 patients were regarded as having combined immunodeficiency in childhood, based on the additional features of autoimmunity or opportunistic infections. During lifetime, twelve patients had received immunoglobulin replacement therapy and one patient had undergone a hematopoietic stem cell transplant. Retrospective laboratory data demonstrated lymphopenia in 36/83 (43%) patients in childhood and in 37/74 (50%) patients in adulthood.

### Live-Vaccine Preventable Diseases in Patients With CHH

A significant number of patients reported history of measles (*n* = 32), mumps (*n* = 24) and/or rubella (*n* = 18) (Table 1), that, in the majority of patients, was also confirmed from the hospital records. All cases occurred in unvaccinated patients, prior to the 1980s, and had been diagnosed clinically. No hospitalizations were linked to measles, mumps, or rubella. Also, no long-term complications have been documented, including no cases of measles-associated subacute sclerosing panencephalitis or rubella-associated granulomatous skin lesions.

**TABLE 1:** Live vaccine preventable disease history, live vaccine immunization history and serological testing in 104 patients with CHH.

**Disease**	**Vaccine**	**Clinical history of disease (*n*)**	**Written evidence of immunization (*n*)**	**Complications post-immunization (*n*)**	**Positive serology^a^ [*n*/tested (%)]**
Measles	Measles	32	12	0	44/50 (88)
Mumps	Mumps	24	NA	NA	33/48 (69)
Rubella	Rubella	18	8	0	40/48 (83)
	MMR	NA	40	0	NA
Polio	Sabin	0	37	0	n/a
Rota virus	Rota virus	n/a	2	0	n/a
Smallpox	Smallpox	0	22	0	n/a
Tuberculosis	BCG	1	78	0	n/a
Varicella	VZV^b^	73	10	1^c^	49/50 (98)^d^
Yellow fever	Yellow fever	0	1	0	n/a

*BCG bacillus Calmette–Guérin vaccine, MMR measles, mumps and rubella combined vaccine, n number of patients, n/a not accessed, NA not applicable, VZV varicella zoster virus. ^*a*^Post-vaccination serology results only were reported in those immunized against varicella. ^*b*^Patients enrolled in the clinical trial were also included. ^*c*^Complications are described in detail under the section *Clinical trial of live varicella zoster virus vaccine in patients with CHH*. ^*d*^Seronegative patient had no history of varicella or VZV vaccine administration, but deceased before enrollment into VZV vaccine clinical trial.*

A history of varicella was reported in 73 patients with CHH. In addition, five more patients were seropositive for VZV despite negative clinical history. Of these 78 patients, seven were hospitalized for varicella due to severe disease, at least two of whom had concomitant pneumonia, all seven recovered. In addition, nine more patients were treated with acyclovir immediately after the onset of rash, which successfully prevented the severe course of varicella, and eight more individuals were administered VZV-specific immunoglobulin after a contact with varicella and did not develop disease.

### Immunization With Live Viral Vaccines in Patients With CHH

Historically, measles vaccine was used in Finland in 1975–1982, rubella vaccine in 1975–1988, and MMR vaccine has been used since 1982. Written evidence of immunization was available in 90/104 patients, and the majority of them (96%, 86/90) have been vaccinated with one or more live vaccines (Table 1). No hospitalizations or serious complications were reported after any live vaccine administration, not even after immunization against smallpox, polio and yellow fever.

For measles-containing vaccines, one patient received the first dose in adulthood, otherwise primary immunization was performed in childhood, at median age of 1.7 years (range 1.1–12.6 years).

Prior to the initiation of our clinical trial, we were aware of five patients with CHH successfully immunized with live VZV vaccine during the second year of life, with no adverse events.

### Serology for Measles, Mumps, Rubella, and Varicella in Patients With CHH

Of the 50 patients tested for antibodies against measles (*n* = 50), mumps (*n* = 48), rubella (*n* = 48), and varicella (*n* = 50) viruses, previous immunization has been documented with MMR (*n* = 22), VZV (*n* = 9), rubella (*n* = 2) and measles (*n* = 1) vaccines.

For varicella, all patients with a history of disease or immunization showed durable humoral response with 100% (44/44) of seropositivity when tested (Table 2). For measles, 100% (15/15) of patients with a history of disease had measurable antibodies, as had 96% (22/23) immunized patients (Table 2). For mumps, seropositivity was observed in 86% (12/14) of patients with a history of disease and in 75% (15/20) of immunized patients (Table 2). For rubella, these proportions were 100% (13/13) and 91% (20/22), respectively (Table 2). In terms of the clinical staging of immunodeficiency, all seronegative patients were asymptomatic, except for two patients with combined immunodeficiency, of whom one did not develop antibodies against measles and the other against mumps after MMR vaccination.

**TABLE 2:** Results of antibody measurements for measles, mumps, rubella (MMR) and varicella zoster viruses from serum samples of patients with cartilage-hair hypoplasia.

**Disease**	**Clinical history**	**Immunized**	**Serology positive [*n*/tested (%)]**
Measles	Yes: 32	Yes: 4	3/3 (100)
		No/no data: 28	12/12 (100)
	No/no data: 72	Yes: 39	19/20 (95)
		No/no data: 34	10/15 (67)
Mumps	Yes: 24	Yes: 2	1/1 (100)
		No/no data: 22	11/13 (85)
	No/no data: 80	Yes: 37	15/20 (75)
		No/no data: 43	6/14 (43)
Rubella	Yes: 18	Yes: 4	2/2 (100)
		No/no data: 14	11/11 (100)
	No/no data: 86	Yes: 43	18/20 (90)
		No/no data: 43	9/15 (60)
Varicella	Yes: 73	Yes: 0	–
		No/no data 73	35/35 (100)
	No/no data: 31	Yes: 10	9/9 (100)
		No/no data: 21	5/6 (83)

Median time between the last dose of measle-containing vaccines and serum sampling for serology was 24.6 years (range 0.3–36.9 years). This median time interval was similar for MMR and for rubella-containing vaccines (24.7 and 24.9 years, respectively). All five patients immunized with live VZV vaccine, had detectable antibodies against VZV in serum 5–19 years (median 17 years) post-immunization.

### Clinical Trial of Live Varicella Zoster Virus Vaccine in Patients With CHH

Table 3 summarizes the results of the clinical trial on live VZV vaccine in five patients with CHH. We immunized four children and one adult, all with clinically silent immunodeficiency. Three patients were lymphopenic, with various abnormalities in lymphocyte subpopulations. Lymphocyte responses to stimulation with mitogens were normal in all patients. All five patients had normal levels of immunoglobulin G.

**TABLE 3:** Summary of the clinical trial of live varicella zoster virus vaccine in patients with cartilage-hair hypoplasia.

**Patient**	**1**	**2**	**3**	**4**	**5**
Age at primary immunization, years	1⋅5	2⋅7	6	16	45
Symptoms of combined immunodeficiency prior to immunization^a^	None	None	None	None	None
Laboratory parameters prior to immunization					
Total lymphocyte count, cells/mm^3^	**2112**	3020	**960**	**1210**	1590
CD3 + T cell count, cells/mm^3^	**1150**	2240	**580**	**810**	1200
CD4 + T cell count, cells/mm^3^	**827**	1482	**294**	543	625
CD8 + T cell count, cells/mm^3^	**140**	530	**110**	**140**	590
CD19 + B cell count, cells/mm^3^	**290**	580	**120**	130	160
CD16/56 + NK cell count, cells/mm^3^	310	**110**	170	90	160
Lymphocyte response to stimulation with PHA	Normal	Normal	Normal	Normal	Normal
Number of vaccine doses	2	2	1	2	1
Time interval between vaccine doses, months	6	6	n/a	4.5	n/a
VZV-specific immunoglobulin G after the first dose	neg	neg	pos	neg	pos
VZV-specific immunoglobulin G after the second dose	pos	pos	NA	pos	NA
Cellular response by elispot after the first dose	neg	pos	pos	pos	pos
Cellular response by elispot after the second dose	pos	n/a	NA	pos	NA
Vaccine adverse events	None	None	None	None	Yes^b^

*Numbers in bold represent cell counts below the normal range for age. n/a not available, NA – not applicable, neg – negative, PHA – phytohemagglutinin, pos – positive, VZV – varicella zoster virus. ^*a*^Including severe or opportunistic infections, autoimmunity, or malignancy. ^*b*^Post-immunization adverse events (rash and knee swelling) are described in detail in text.*

None of the four immunized children developed adverse events. All five patients are well 2.8–4.5 years post-immunization, and no breakthrough varicella has been reported. Patient 5 reported a self-limited upper respiratory tract infection two weeks post-immunization. He then developed a rash during the third week post-immunization when few painless yellow-reddish round papules and ulcers (but not vesicles) 0.5 cm in size appeared on his abdomen, neck, and head within several days. The rash persisted unchanged for a week and then gradually disappeared. In addition, the patient developed swelling and morning stiffness in his right knee three weeks post-immunization, which did not disturb his walking and cycling. The patient declined antiviral and anti-inflammatory therapy and reported no complaints on the follow-up seven weeks post-immunization. There was no viremia at the time of rash, confirmed by negative blood VZV nucleic acid testing.

**FIGURE 2:**
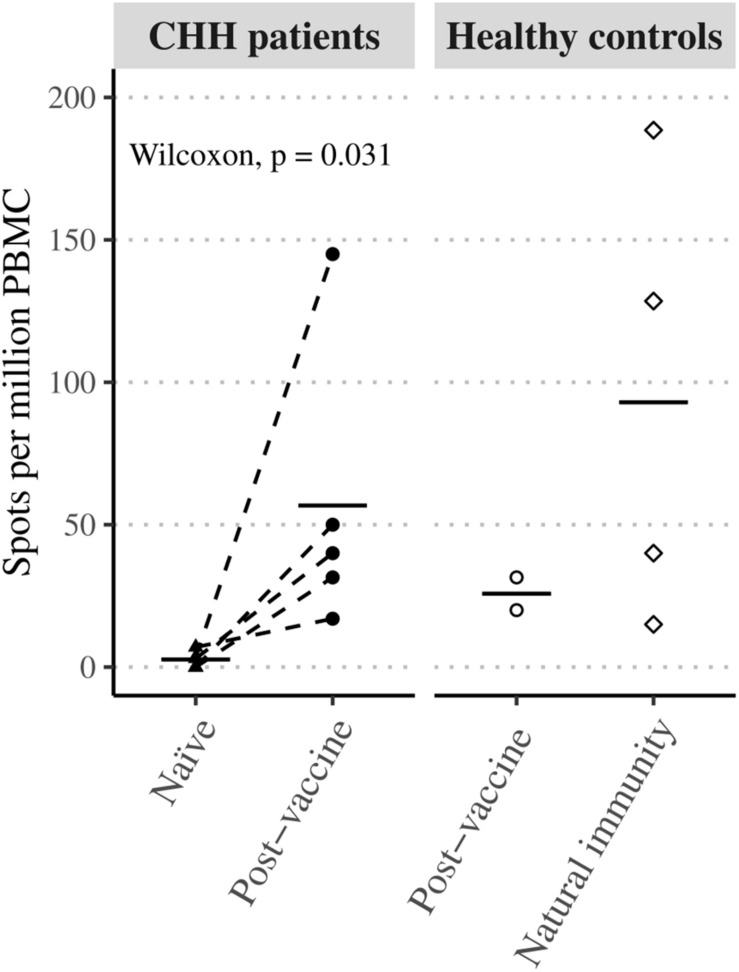
Varicella zoster virus antigen response in CHH patient samples. Peripheral blood mononuclear cells of naïve and post-vaccine paired patient samples showed statistically significant induction of cellular immunity in interferon-γ elispot antigen assay. The level of induction (mean, black lines) was similar to a single healthy control.

Three patients who did not develop measurable humoral response after the first vaccine dose demonstrated seroconversion after the second immunization. All patients also demonstrated the development of cellular response post-immunization when measured by elispot, 4/5 after the first vaccine dose (Figure 2).

## Discussion

This is the first study describing the administration of live viral vaccines in a large cohort of patients with CHH. We demonstrate the safety and efficacy of MMR and VZV vaccines in this patient population, based on the retrospective data on provider-recorded immunizations, absence of breakthrough infections or hospitalizations linked to immunizations, as well as the durable serologic response. We also present the results of a clinical trial of live VZV vaccine in patients with CHH, which showed the generation of both humoral and cellular immune responses. Among the participants of our clinical trial, the incidence of varicella-like post-vaccination rash was 20% (1/5), compared with the reported rates of 0–7% in healthy individuals (22).

Prior to the 1980s, when MMR immunization was introduced in Finland, many patients with CHH had contracted MMR-diseases with uneventful course. This is in line with the generally milder immunodeficiency phenotype in Finnish individuals with CHH who rarely develop severe opportunistic infections (21). However, varicella clearly is an exception, as many patients with CHH suffer from prolonged rash and fever and some develop pneumonia and require hospitalization (13). Many patients with CHH are pre-emptively treated with acyclovir and given VZV immunoglobulin for the post-exposure prophylaxis. Immunization against VZV may obviate the need for prophylaxis, antiviral treatment and hospitalizations. The same considerations can be applied to the MMR vaccine.

Patients with CHH mount robust humoral immune response to measles and varicella (both natural infection and immunization), which remains measurable for decades. Antibodies to mumps either do not develop or wane over time in a significant proportion (14–25%) of patients with CHH, raising the concern about insufficient immunity against mumps. Also, rarely, patients with CHH may not develop seropositivity for rubella after MMR administration. Therefore, antibody measurement should be routinely used to assess vaccination responses in patients with CHH and booster doses should be given in case of insufficient immunity.

The pattern of seropositivity to MMR and VZV in patients with CHH are similar to those in healthy individuals, with good responses to measles, varicella and rubella, but lower rates of seropositivity to mumps (23, 24). However, in our clinical trial of VZV vaccine, 60% (3/5) of participants failed to develop antibodies against VZV after the single vaccine dose, while in healthy children this proportion is 24% (25).

This is the first study describing the production of IFN-γ in stimulated PBMC from patients with CHH. Our assessment of cellular response to VZV vaccine demonstrate that patients with CHH develop cellular immunity probably comparable to healthy immunized controls. The comparison was problematic due to the small number of healthy immunized controls. Antibodies are essential to neutralize pathogens in extracellular space. However, cellular immunity is critical for controlling and containing intracellular pathogens, especially viruses with the ability to form latency, such as VZV. Thus, the protection against VZV is provided by multitude of simultaneously acting immunological mechanisms (26). Here, we observed clear cellular responses in two CHH patients after the first vaccine dose despite no development of antibodies to VZV. This may be explained by delayed antibody production consistent with combined immunodeficiency in CHH. The activation of cellular response against VZV in CHH patients suggests that these individuals may have sufficient immune protection against VZV despite the decreased humoral response. Our data support the recommendation of two doses of VZV vaccine, not single dose, to ensure optimal immune response of both, humoral and cellular, arms of the immune system.

Although there were no reported long-term consequences of live vaccines in our cohort, clinicians and patients should be aware of the risk of delayed development of vaccine-strain rubella virus-associated skin granulomas in patients with CHH (27). In addition, there were no reactivation of vaccine-strain VZV in our patients, possibly reflecting the effective cellular immunity against VZV. However, such a reactivation, occurring even years after primary immunization, and accompanied by central nervous system infection, has been described, both in immunocompetent and immunocompromised adolescents (28).

Laboratory parameters do not correlate with clinical severity or prognosis in CHH (21), and our study reports successful immunization of lymphopenic patients, therefore mild lymphopenia itself should not be an absolute contraindication for live vaccine administration in CHH. Clinicians can utilize laboratory criteria used in the management of patients with human immunodeficiency virus infection or patients with partial DiGeorge syndrome to make decisions on immunization in patients with CHH. Importantly, all vaccinated patients in our clinical trial demonstrated normal lymphocyte proliferation responses to mitogens prior to immunization. Immunization may be considered in clinically asymptomatic patients with CHH or those with mild recurrent respiratory tract infections and no other clinical features of immunodeficiency. However, individuals considered for the immunization should be selected cautiously and the qualitative assessment of lymphocyte subsets and function should be performed prior to immunization. Even though several patients with clinical features of combined immunodeficiency have been immunized with MMR successfully, we advise against live vaccine administration to these patients.

The strengths of our study are (1) the large number of patients for whom clinical and laboratory data were available, (2) written evidence of immunization in the majority of the study participants, (3) long-term follow-up data and assessment of serologic response to immunization in the long-term, as well as (4) assessment of both, humoral and cellular immune responses in a clinical trial. The drawbacks of our clinical trial include small sample size and relatively short follow-up time, calling for further studies with longer follow-up to evaluate the efficacy and long-term safety of VZV vaccine.

In conclusion, we provide retrospective and prospective data on the safety and efficacy of live viral vaccines in Finnish patients with CHH. Immunization with live vaccines should be considered in selected CHH patients with clinically mild immunodeficiency.

## Data Availability Statement

All datasets presented in this study are included in the article/supplementary material.

## Ethics Statement

The studies involving human participants were reviewed and approved by Institutional Ethics Committee at the Helsinki University Hospital and University of Helsinki, Finland. Written informed consent to participate in this study was provided by the participants’ legal guardian/next of kin.

## Author Contributions

SV, PK, and OM planned the study. SV, PK, LK, and MG carried out a clinical trial. JH and HS prepared PBMC for further analysis. EK and IK planned and performed the elispot analysis. SV drafted the manuscript. All authors contributed to the writing and critical review of the manuscript and approved the final version.

## Conflict of Interest

The authors declare that the research was conducted in the absence of any commercial or financial relationships that could be construed as a potential conflict of interest.

## Abbreviations

CHH: cartilage-hair hypoplasia
IFN-γ: interferon gamma
MMR: measles, mumps, rubella
PBMC: peripheral blood mononuclear cells
VZV: varicella oster virus.
